# Determining the risk-factors for molecular clustering of drug-resistant tuberculosis in South Africa

**DOI:** 10.1186/s12889-023-17234-x

**Published:** 2023-11-24

**Authors:** Halima Said, Elizabeth Kachingwe, Yasmin Gardee, Zaheda Bhyat, John Ratabane, Linda Erasmus, Tiisetso Lebaka, Minty van der Meulen, Thabisile Gwala, Shaheed Omar, Farzana Ismail, Nazir Ismail

**Affiliations:** 1 Centre for Tuberculosis, National Institute of Communicable Diseases, Moderfontein Road, Sandringham, Johannesburg, code 2131 South Africa; 2 Centre for Enteric Diseases, National Institute of Communicable Diseases, Sandringham, Johannesburg, South Africa; 3 Division of Surveillance and Outbreak Response, National Institute of Communicable Diseases, Sandringham, Johannesburg, South Africa

**Keywords:** Clustering, Transmission, Risk-factors, Drug resistant TB, South Africa

## Abstract

**Background:**

Drug-resistant tuberculosis (DR-TB) epidemic is driven mainly by the effect of ongoing transmission. In high-burden settings such as South Africa (SA), considerable demographic and geographic heterogeneity in DR-TB transmission exists. Thus, a better understanding of risk-factors for clustering can help to prioritise resources to specifically targeted high-risk groups as well as areas that contribute disproportionately to transmission.

**Methods:**

The study analyzed potential risk-factors for recent transmission in SA, using data collected from a sentinel molecular surveillance of DR-TB, by comparing demographic, clinical and epidemiologic characteristics with clustering and cluster sizes. A genotypic cluster was defined as two or more patients having identical patterns by the two genotyping methods used. Clustering was used as a proxy for recent transmission. Descriptive statistics and multinomial logistic regression were used.

**Result:**

The study identified 277 clusters, with cluster size ranging between 2 and 259 cases. The majority (81.6%) of the clusters were small (2–5 cases) with few large (11–25 cases) and very large (≥ 26 cases) clusters identified mainly in Western Cape (WC), Eastern Cape (EC) and Mpumalanga (MP). In a multivariable model, patients in clusters including 11–25 and ≥ 26 individuals were more likely to be infected by Beijing family, have XDR-TB, living in Nelson Mandela Metro in EC or Umgungunglovo in Kwa-Zulu Natal (KZN) provinces, and having history of imprisonment. Individuals belonging in a small genotypic cluster were more likely to infected with Rifampicin resistant TB (RR-TB) and more likely to reside in Frances Baard in Northern Cape (NC).

**Conclusion:**

Sociodemographic, clinical and bacterial risk-factors influenced rate of *Mycobacterium tuberculosis* (*M. tuberculosis*) genotypic clustering. Hence, high-risk groups and hotspot areas for clustering in EC, WC, KZN and MP should be prioritized for targeted intervention to prevent ongoing DR-TB transmission.

## Background

Drug-resistant tuberculosis (DR-TB) is a growing threat to global TB control efforts. The burden of DR-TB in high-burden countries is largely driven by transmission of those strains. Understanding factors driving DR-TB transmission and interventions aimed at reducing transmission may be critical for successful control of the DR-TB epidemic in these settings. Furthermore, addressing the heterogeneity of DR-TB transmission is important, as there is a wide geographical variation in disease burden within and between settings as well as localized transmission in subpopulation.

Molecular epidemiological studies have been useful in a number of countries in supporting TB control by identifying drivers for transmission. These studies have shown that patient-related risk [1, 2], environment [2, 3] and bacterial factors influence TB transmission [4]. However there are varying findings on risk-factors for clustering between studies, particularly between the low-incidence and high incidence countries [5, 6]. In low-incidence countries, risk-factors such as alcohol and drug abuse, immigrant status, homelessness, urban residence, and young age are the major risk-factors influencing clustering [7–11]. Whereas, in lower-middle income countries, information on risks-factors is scarce. Only few studies in high-TB incidence countries have assessed the risk-factors for clustering. The risk-factors identified in these studies include age [12], prior imprisonment [13], treatment failure, visitation of social settings such as bars and churches [14] as well as Human Immunodeficiency Virus (HIV) infection [15–17].

South Africa (SA) has one of the highest burdens of DR-TB in the world. The prevalence of DR-TB varies greatly across different provinces, with majority of DR-TB cases in Kwa-Zulu Natal (KZN), Western Cape (WC), Eastern Cape (EC) and Gauteng (GP) [18]. This variation in burden of the DR-TB could be due to varying distribution of individual and community level risk-factors, and variations in TB control programme performance. Thus, a better understanding of risk-factors for clustering can help to direct resources and efforts to specifically targeted high-risk groups as well as areas that contribute disproportionately to transmission.

This study aimed to identify the potential risk-factors driving DR-TB transmission in SA, using data collected from a sentinel molecular surveillance of Rifampicin-Resistant-TB (RR-TB) which was conducted between 2014 and 2018. In addition, we aimed to describe the characteristics of cases by cluster size and investigate whether risk-factors vary by cluster size.

## Methods

### Study population and setting

The study used retrospective data from sentinel molecular surveillance of RR-TB. The study included culture-positive samples from patients newly diagnosed with RR-TB via Xpert *M. tuberculosis*/RIF or Xpert *M. tuberculosis*/RIF Ultra assay between 2014 and 2018. The surveillance was implemented at eight of the nine provinces, with at least one district targeted per province. These provinces included: Nelson Mandela Metro (EC), Frances Baard (Northern Cape [NC]), Ehlanzeni (Mpumalanga [MP]), Dr Kenneth Kaunda (North West [NW]) and Umgungunglovo (KZN), City of Johannesburg (GP), Mangauang (Free State [FS]), City of Cape Town Metro, Cape Winelands and West Coast (WC). All RR-TB samples were submitted to the Center for TB (CTB), at the National Institute for Communicable Diseases (NICD), in Johannesburg for culture and genotyping. All culture confirmed samples were genotyped by combination of spoligotyping and 24-loci MIRU-VNTR typing. Said et al. (19) provides a detailed description of the study’s design, study population, and laboratory [19].

### Cluster definition

Clustered cases were defined as two or more patients having identical patterns by both spoligotyping and 24-loci MIRU-VNTR typing. A non-clustered (unique) case was defined as any case from the study population having a unique pattern not shared by any other case.

Multi-drug-resistant (MDR) TB was defined as resistance to at least isoniazid (INH) and rifampicin (R); while extensively drug-resistant (XDR) TB was defined as MDR-TB with additional resistance to any fluoroquinolone (FLQ) and to at least one of the three injectable second-line drugs: amikacin (AMK), kanamycin (KAN) and/or capreomycin (CAP).

### Analysis

Descriptive statistics were used to present the number and proportion of clustered strains, non-clustered strains, clusters and distribution of cluster size. We defined the size of a cluster by categorising cases into four groups: 2–5 cases per cluster [small cluster], 6–10 cases per cluster [medium cluster], 11–25 cases per cluster [large cluster], and ≥ 26 cases [very large cluster].

We investigated risk-factors for cases belonging to molecular clusters of different sizes. Our outcome of interest is a categorical variable with five levels, therefore multinomial logistic regression which is an extension of the simple logistic regression was used. For each risk factor, an odds ratio (OR) was calculated for clustered cases (four cluster size outcomes) and cases not in a cluster formed a comparison group. Risk-factors were investigated at single-variable analysis and variables with an association of p < 0.2 were included in the initial multivariable model. The final multivariable model was built by stepwise backward elimination of variables which did not contribute significantly to produce a final model. A p-value of 0.05 was used as threshold. For each risk factor, an OR was calculated for clustered cases (four cluster size outcomes) and cases not in a cluster formed a comparison group.

Exposure variables (from questionnaire) included: demographic (age, sex, income, and province), clinical characteristics (previous treatment and HIV status), high-risk work settings for transmission (health care worker and mine workers) and laboratory finding (sputum smear result and drug susceptibility profile).

## Result

During the 5-years study period, a total of 374,399 TB cases were reported by the TB surveillance program for the ten districts in 8 provinces included in the study. The TB surveillance program reports only laboratory confirmed TB cases which is based on a positive TB result for either an Xpert MTB/Rif or Xpert MTB/ultra assay, culture, line Probe assay or smear microscopy. Of the 374,399 cases, 17,399 were RR-TB (3365 from Nelson Mandela Metro in EC, 919 from Mangauang in FS, 4042 from City of Johannesburg in GP, 1533 from Umgungunglovo in KZN, 2798 from Ehlanzeni in MP, 1383 from Dr Kenneth Kaunda in NW, 605 from Frances Baard in NC and 2754 from three districts in WC). The current study is a sentinel surveillance and included only patients who provided written informed consent and a second sputum sample for the study. A total of 2893 culture confirmed RR-TB cases had genotyping results which is 17% of the reported RR-TB cases in the 10 districts.

Of the 2893 with genotyping results, 864 (29.9%) were collected from the three district in WC, 696 (24.1%) were from Nelson Mandela Metro, 419 (14.5%) were from Ehlanzeni, 343 (11.9%) were from Dr Kenneth Kaunda, 224 (7.7%) were from Umgungunglovo, 138 (4.8%) were from City of Johannesburg, 132 (4.6%) were from Frances Baard and 76 (2.6%) were from Mangauang. For one (0.03%) isolate, no information on province was available.

Strain families based on spoligotyping could be assigned to 2752 (95.1%) cases. The most common lineage was Beijing family identified (1432/2752,52.0%), followed by LAM (323/2752,11.7%), T (263/2752,9.6%), EAI (208/2752,7.6%), S (172/2752,6.3%), X (204/2752,7.4%), H (86/2752,3.1%). The remaining 2.3% (64/2752) isolates belonged to other genotype families.

A total of 51.8% (1498/2893) of the isolates belonged to molecular clusters. A total of 277 clusters were identified, with cluster size ranging from two to 259 isolates. Most clusters (226/277,81.6%) were small (2–5 cases), 10.8% (30/277) were medium sized (6–10 cases), 13/278 (4.7%) were large (11–25 cases) and 2.9% (8/277) were very large with 26–259 cases.

### Characteristics of study population

Questionnaire data was available for all the provinces with the exception of WC. The characteristics of patients for the seven provinces is summarized in Table 1. For WC, only demographics (age and gender) and laboratory test results (sputum smear status and drug susceptibility testing) were available from the laboratory information system (Table 2).

**Table 1:** Characteristics of DR-TB cases in molecular clusters of different sizes in seven high-burden districts

Characteristics	All cases (N = 2029)	Not clustered (N = 1159)	Clustered (N = 870)	Cases in cluster n (%)			
				2 − 5	6–10	11–25	≥ 26
**Sex**							
Male	1100 (100)	629 (57.2)	471 (42.8)	222 (47.1)	77 (16.3)	35 (7.4)	137 (29.1)
Female	856 (100)	486 (56.8)	370 (43.2)	173 (46.8)	70 (18.9)	21 (5.7)	106 (28.6)
Unknown	58 (100)	43 (58.6)	24 (41.4)	15 (62.5)	2 (8.3)	6 (25.0)	1 (4.2)
**Age group**							
18–44	1482 (100)	848 (57.2)	634 (42.8)	299 (47.2)	105 (16.6)	45 (7.1)	185 (29.2)
45–64	461 (100)	264 (57.3)	197 (42.7)	94 (47.7)	38 (19.3)	16 (8.1)	49 (24.9)
=>65	39 (100)	24 (61.5)	15 (38.5)	7 (46.7)	3 (20.0)	0 (0.0)	5 (33.3)
Unknown	47 (100)	23 (48.9)	24 (51.1)	13 (54.2)	4 (16.7)	2 (8.3)	5 (20.8)
**Province**							
Eastern Cape	696 (100)	283 (40.7)	413 (59.3)	110 (26.6)	68 (16.5)	39 (9.4)	196 (47.5)
Free State	76 (100)	63 (82.9)	13 (17.1)	13 (100)	0 (0.0)	0 (0.0)	0 (0.0)
Gauteng	138 (100)	98 (71.0)	40 (29.0)	31 (77.5)	9 (22.5)	0 (0.0)	0 (0.0)
Kwazulu-Natal	224 (100)	137 (61.2)	87 (38.8)	54 (62.1)	21 (24.1)	12 (13.8)	0 (0.0)
Mpumalanga	419 (100)	235 (56.1)	184 (43.9)	98 (53.3)	26 (14.1)	12 (6.5)	48 (26.1)
Northern Cape	132 (100)	110 (83.3)	22 (16.7)	22 (100)	0 (0.0)	0 (0.0)	0 (0.0)
North West	343 (100)	232 (67.6)	111 (32.4)	85 (76.6)	0 (0.0)	0 (0.0)	0 (0.0)
Unknown	1 (100)	1 (100)	0 (0.0)	0 (0.0)	0 (0.0)	0 (0.0)	0 (0.0)
**Income**							
No	1245 (100)	693 (55.7)	552 (44.3)	256 (46.4)	90 (16.3)	44 (8.0)	162 (29.3)
Yes	448 (100)	254 (56.7)	194 (43.3)	87 (44.8)	40 (20.6)	13 (6.7)	54 (27.8)
Unknown	336 (100)	212 (63.1)	124 (36.9)	70 (56.5)	20 (16.1)	6 (4.8)	28 (22.6)
**Occupation**							
Healthcare	53 (100)	32 (60.4)	21 (39.6)	8 (38.1)	6 (28.6)	3 (14.3)	4 (19,0)
Mine	90 (100)	65 (72.2)	25 (27.8)	17 (68.0)	5 (20.0)	2 (8.0)	1 (4.0)
Other/unemployed	1530 (100)	841 (55.0)	689 (45.0)	316 (45.9)	116 (16.8)	50 (7.3)	207 (30.0)
Unknown	356 (100)	221 (62.1)	135 (37.9)	72 (53.3)	23 (17.0)	8 (5.9)	32 (23.7)
**HIV status**							
Negative	437 (100)	218 (49.9)	219 (50.1)	96 (43.8)	30 (13.7)	17 (7.8)	76 (34.7)
Positive	1223 (100)	707 (57.8)	516 (42.2)	240 (46.5)	99 (19.2)	40 (7.8)	137 (26.6)
Unknown	369 (100)	234 (63.4)	135 (36.6)	77 (57.0)	21 (15.6)	6 (4.4)	31 (23.0)
**Previous treatment**							
No	800 (100)	439 (54.9)	361 (45.1)	186 (51.5)	54 (15.0)	25 (6.9)	96 (26.6)
Yes	868 (100)	489 (56.3)	379 (43.7)	153 (40.4)	76 (20.1)	32 (8.4)	118 (31.1)
Unknown	361 (100)	231 (64.0)	130 (36.0)	74 (56.9)	20 (15.4)	6 (4.6)	30 (23.1)
**Imprisonment**							
No	1537 (100)	865 (56.3)	672 (43.7)	312 (46.4)	120 (17.9)	48 (7.1)	192 (28.6)
Yes	142 (100)	76 (53.5)	66 (46.5)	29 (43.9)	9 (13.6)	9 (13.6)	19 (28.8)
Unknown	350 (100)	218 (62.3)	132 (37.7)	72 (54.5)	21 (15.9)	6 (4.5)	33 (25.0)
**Occupation**							
Healthcare	53 (100)	32 (60.4)	21 (39.6)	8 (38.1)	6 (28.6)	3 (14.3)	4 (19.0)
Mine	90 (100)	65 (72.2)	25 (27.8)	17 (68.0)	5 (20.0)	2 (8.0)	1 (4.0)
Other/unemployed	1530 (100)	841 (55.0)	689 (45.0)	316 (45.9)	116 (16.8)	50 (7.3)	207 (30)
Unknown	356 (100)	221 (62.1)	135 (37.9)	72 (53.3)	23 (17.0)	8 (5.9)	32 (23.7)
**Smear Result**							
1+	251 (100)	154 (61.4)	97 (38.6)	50 (51.5)	15 (15.5)	8 (8.2)	24 (24.7)
2+	320 (100)	188 (58.8)	132 (41.3)	56 (42.4)	17 (12.9)	8 (6.1)	51 (38.6)
3+	831 (100)	451 (54.3)	380 (45.7)	174 (45.8)	70 (18.4)	27 (7.1)	109 (28.7)
Negative	587 (100)	340 (57.9)	247 (42.1)	123 (49.8)	47 (19.0)	19 (7.7)	58 (23.5)
Unknown	2 (100)	1 (50.0)	1 (50.0)	0 (0.0)	0 (0.0)	0 (0.0)	0 (0.0)
**DST**							
INH-R/SUS	194 (100)	120 (61.9)	74 (38.1)	51 (68.9)	9 (12.2)	1 (1.4)	13 (17.6)
RIF-R	496 (100)	334 (67.3)	162 (32.7)	88 (54.3)	26 (16.0)	14 (8.6)	34 (21.0)
MDR	1133 (100)	637 (56.2)	496 (43.8)	243 (49.0)	89 (17.9)	42 (8.5)	122 (24.6)
XDR	175 (100)	50 (28.6)	125 (71.4)	23 (18.4)	22 (17.6)	6 (4.8)	74 (59.2)
Unknown	31 (100)	18 (58.1)	13 (41.9)	8 (61.5)	4 (30.8)	0 (0.0)	1 (7.7)
**Strains**							
Beijing	856 (100)	371 (43.3)	485 (56.7)	170 (35.1)	80 (16.5)	39 (8.0)	196 (40.4)
EAI	202 (100)	101 (50.0)	101 (50.0)	35 (34.7)	6 (5.9)	12 (11.9)	48 (47.5)
LAM	271 (100)	178 (65.7)	93 (34.3)	67 (72.0)	14 (15.1)	12 (12.9)	0 (0.0)
Other	700 (100)	509 (72.7)	191 (27.3)	141 (73.8)	50 (26.2)	0 (0.0)	0 (0.0)

**Table 2:** Single and multiple variable multinomial logistic regression analysis for risk-factors associated with cases in a molecular cluster of different sizes in South Africa (2014–2018)

	**Single Variable Multinomial regression**				Multivariate Multinomial regression			
	2–5	6–10	11–25	≥ 26	2–5	6–10	11–25	≥ 26
**Sex**								
Male	1	1	1	1				
Female	1.01 (0.80–1.27)	1.18 (0.34–1.66)	0.78 (0.45–1.35)	0.10 (0.75–1.32)				
**Age group**								
18–44	1	1	**1**	1				
45–64	1.00 (0.77–1.32)	1.16 (0.78–1.73)	1.14 (0.63–2.05)	0.85 (0.60–1.20)				
>=65	0.83 (0.35–1.94)	1.00 (0.30–3.41)	-	0.95 (0.36–2.53)				
Unknown	1.60 (0.80–3.20)	1.40 (0.48–4.14)	1.64 (0.37–7.17)	1.10 (0.37–2.66)				
**Province**								
Eastern Cape	0.93 (0.67–1.29)	2.17 (1.31–3.52)*	2.70 (1.38–5.27)*	3.39 (2.36–4.86)*	0.80 (0.57–1.34)	1.52 (0.89–2.57)	5.14 (2.07–12.76)*	6.53 (3.46–12.35)*
Free State	0.49 (0.26–0.94)*	-	-	-	0.49 (0.25–3.96)	-		**-**
Gauteng	0.76 (0.47–1.12)	0.83 (0.37–1.83)	-	-	0.70 (0.43–1.15)	0.91 (0.40–2.07)	**-**	**-**
Kwazulu-Natal	0.95 (0.63–1.40)	1.39 (0.75–2.56)	1.72 (0.75–3.92)	-	0.97 (0.64–1.47)	1.42 (0.74–2.71)	**-**	**-**
Northern cape	0.47 (0.28–0.80)*	-	-	-	0.51 (0.29–0.87)*	-	5.52 (2.00-15.33)*	**-**
Mpumalanga	1	1	1	1	1	1	1	1
North West	0.88 (0.62–1.24)	1.01 (0.57–1.80)	-	-	0.95 (0.66–1.37)	1.17 (0.63–2.17)	**-**	**-**
**Occupation**								
Other	1	1	1	1	1	1	1	1
Healthcare	0.67 (0.30–1.46)	1.36 (0.55–3.32)	1.57 (0.47–5.32)	0.51 (0.18–1.45)	0.73 (0.33–1.62)	1.42 (0.56–3.61)	1.61 (0.44–5.91)	0.52 (0.16–1.73)
Mine	0.69 (0.40–1.21)	0.56 (0.4–1.21)	0.52 (0.12–2.17)	0.06 (0.01–0.45)*	0.73 (0.41–1.29)	0.79 (0.30–2.10)	1.02 (0.21–5.03)	0.12 (0.01-1.00)
Unknown	0.87 (0.64–1.17)	0.75 (0.47–1.21)	0.61 (0.28–1.30)	0.59 (0.39–0.88)*	2.00 (0.43–1.86)	1.39 (0.46–4.24)	3.14 (0.74–13.39)	0.62 (0.20–1.87)
**Income** .								
No	1	1	1	1				
Yes	0.92 (0.70–1.23)	1.21(0.81–1.81)	0.81 (0.43–1.52)	0.91 (0.65–1.28)				
Unknown	0.89 (0.66–1.21)	0.73(0.44–1.21)	0.45 (0.19–1.06)	0.56 (0.37–0.87)*				
**Imprisonment**								
No	1	1	1	1	1	1	1	
Yes	1.06 (0.68–1.65)	0.85 (0.42–1.75)	2.13 (1.00-4.52)*	1.13 (0.67–1.91)	1.07 (0.74–1.85)	0.94 (0.45–1.98)	3.24 (1.39–7.51)*	
Unknown	0.92 (0.68–1.23)	0.69 (0.43–1.13)	0.50 (0.21–1.17)	0.68 (0.46–1.02)	1.07 (0.51–2.23)	0.58 (0.18–1.86)	0.39 (0.08–1.95)	
**HIV status**								
Negative	1	1	1	**1**				
Positive	0.77 (0.58–1.02)*	1.01 (0.66–1.57)	0.73 (0.40–1.31)	0.56 (0.40–0.76)*				
Unknown	0.75 (0.53–1.06)*	0.65 (0.36–1.17)	0.33 (0.13–0.85)	0.38 (0.24–0.60)*				
**Previous Treatment**								
**No**	1	1	1	1				
Unknown	0.76 (0.55–1.03)	0.70 (0.41–1.20)	0.46 (0.18–1.13)*	0.59 90.38–0.92)*				
Yes	0.74 (0.58–0.95)	1.26 (0.87-1..83)	1.15 (0.67–1.97)*	1.10 (0.82–1.49)*				
**Sputum smear**								
Negative	1	1	1	1				
1+	0.90 (0.61–1.31)	0.70 (0.38–1.30)	0.93 (0.40–2.17)	0.91 90.55–1.52)				
2+	0.82 (0.57–1.18)	0.65 (0.37–1.17)	0.76 (0.33–1.77)	1.59 (1.04–1.52)*				
3+	1.07 (0.81–1.40)	1.12 (0.76–1.67)	1.07 (0.59–1.96)	1.42 (1.00-2.01)*				
Scanty	1.00 (0.45–2.19)	0.29 (0.04–2.19)	0.72 (0.09–5.57)	0.47 (0.11–2.03)				
**DST**								
SUS/ INH-R	1	1	1	1	1	1	1	1
MDR/ Pre-XDR	0.90 (0.63–1.29)	1.87 (0.91–3.80)	7.91 (1.08–58.04)	1.77 (1.00-3.23)	0.89 (0.62–1.29)	1.96 (0.95–4.05)	6.46 (0.83–50.39)	1.13 (0.55–2.32)
RIF-R	0.62 (0.41–0.93)*	1.04 (0.47–2.28)	5.03 (0.65–38.66)	0.94 (0.48–1.84)	0.60 (0.40–0.91)*	1.07 (0.48–2.37)	5.23 (0.64–42.73)	0.70 (0.32–1.54)
XDR	1.08 (0.60–1.96)	5.87 (2.53–13.63)*	14.40 (1.69–122.70)*	**13.66 (6.95–26.84**)*	0.98 (0.54–1.80)	4.97 (2.08–11.89)*	7.24 (0.79–66.08)	5.08 (2.26–11.40)*
**Strains**								
EAI	1	1	1	1	1	1	1	1
BEIJING	1.32 (0.86–2.02)	3.63 (1.53–8.56)	0.88 (0.45–1.75)	1.11 (0.75–1.63)	1.10 (1.00-2.53)*	3.34 (1.34–8.32)*	0.32 (0.12–0.82)*	0.23 (0.12–0.46)*
LAM	1.09 (0.67–1.75)	1.32 (0.49–3.55)	0.57 (0.25–1.30)	-	1.27 (0.77–2.11)	1.40 (0.50–3.91)	0.37 (0.13–1.04)	**-**
Other	1.00 (0.52–1.23)	1.65 90.69–3.96)	-	-	0.92 (0.58–1.46)	1.83 (0.73–4.55)	-	**-**

*P-value < 0.05

### Characteristics of patients from the seven provinces

The age of patients enrolled in the surveillance ranged from 18 to 89 years (interquartile range (IQR): 29; 45). Over a half 1100/1956 (56.2%) of all cases were males. The majority 1245/1693 (73.5%) of patients with known occupational status were not in employment. Of those with employment, 11.8% work in health care system and 20.1% patients work in mines. Information on previous history of TB was available for 82.2% of cases; of these 52.0% had been previously treated for TB. Information on HIV testing was known for 81.8% of cases; of these 73.7% were HIV positive.

Sputum smear results were available for 99.8%, of which over a half (71.1%) were smear positive. Drug susceptibility testing (DST) for at least INH and RIF was available for 98.5% of the isolates. MDR represented over half of the resistant strains (56.7%).

### Characteristics of patients from three districts in Western Cape

The proportion of males was higher (60.2%) than females (37.5%), while the sex for 0.2% of the patients were not available. The age ranged from 18 to 77 years (IQR: 29; 45). Sputum smear results were available for 89.0%, of which 50.1% were smear positive. Drug susceptibility testing data was available for 99.4% of the isolates. Of those, the majority (69.8%) of the isolates were MDR-TB (Table 3).

**Table 3:** Characteristics of RR-TB cases in molecular clusters of different sizes and single-variable multinomial logistic regression analysis for risk-factors associated with cases in a molecular cluster of different sizes in Western Cape (2014–2018)

	All cases	Not clustered	Clustered	Cases in cluster n (%)				Single Variable Multinomial regression			
	N = 864	N = 236	N = 661	2 − 5	6–10	11–25	≥ 26	2 − 5	6–10	11–25	≥ 26
**Sex**											
Male	520 (60.2)	140 (26.9)	380 (73.1)	110 (28.9)	50 (13.2)	29 (7.6)	191 (50.3)	1	1	1	1
Female	342 (37.5)	95 (27.8)	247 (72.2)	58 (23.5)	31 (12.6)	27 (10.9)	131 (53.0)	0.8 (0.52–1.17)	0.9 (0.54–1.53)	1.4 (0.76–2.46)	1.0 (0.72–1.42)
Unknown	2 (0.2)	1 (50.0)	1 (50.0)	0 (0.0)	0 (0.0)	1 (50.0)	0 (0.0)				
**Age group**											
18–44	643 (74.4)	174 (27.1)	485 (72.9)	130 (27.7)	61 (13.0)	41 (8.7)	237 (50.5)	1	1	1	1
45–64	206 (23.8)	59 (28.6)	147 (71.4)	35 (23.8)	18 (12.2)	14 (9.5)	80 (54.4)	0.8 (0.49–1.27)	0.9 (0.47–1.59)	1.0 (0.51–1.97)	1.0 (0.67–1.46)
=>65	15 (1.7)	3 (20.0)	12 (80.0)	3 (25.0)	2 (16.7)	2 (16.7)	5 (41.7)	1.3 (0.26–6.7)	1.9 (0.31–11.65)	2.8 (0.45–17.48)	1.2 (0.29–5.18)
**Sputum smear**											
Negative	431 (49.9)	126 (29.2)	325 (70.8)	95 (31.1)	33 (10.8)	20 (6.6)	157 (51.5)	1	1	1	1
Scanty	64 (7.4)	17 (26.6)	49 (73.4)	8 (19.1)	5 (10.6)	8 (17.0)	26 (55.3)	0.6 (0.26–1.50)	1.1 (0.38–3.26)	2.9 (1.13–7.77)*	1.2 (0.63–2.36)
1	70 (8.1)	19 (27.1)	55 (72.9)	12 (23.5)	8 (15.7)	4 (7.8)	27 (52.9)	0.8 (0.38–1.80)	1.6 (0.64–3.99)	1.3 (0.40–4.30)	1.1 (0.60–2.14)
2	80 (9.3)	15 (18.8)	67 (81.3)	15 (23.1)	7 (10.8)	9 (13.8)	34 (52.3)	1.3 (0.61–2.84)	1.8 (0.67–4.72)	3.8 (1.45–9.78)*	1.8 (0.94–3.48)
3	124 (14.4)	34 (27.4)	93 (72.6)	17 (18.9)	20 (22.2)	8 (8.9)	45 (50.0)	0.7 (0.34–1.25)	2.2 (1.14–4.39)*	1.5 (0.60–3.65)	1.1 (0.64–1.76)
Unknown	95 (11.0)	25 (26.3)	72 (73.7)	21 (30.0)	8 (11.4)	8 (11.4)	33 (47.1)				
**DST**											
RIF-R	215 (24.9)	96 (44.7)	126 (55.3)	51 (42.9)	13 (10.9)	27 (22.7)	28 (23.5)	1	1	1	1
MDR/pre-XDR	603 (69.8)	124 (20.6)	421 (79.4)	113 (23.6)	62 (12.9)	25 (5.2)	279 (58.2)	1.7 (1.12–2.62)*	3.7 (1.91–7.10)*	0.7 (0.39–1.31)	7.7 (4. 81-12.35)*
XDR	41 (4.7)	13 (31.7)	28 (68.3)	4 (14.3)	6 (21.4)	5 (17.9)	13 (46.4)	0.6 (0.17–1.86)	**3.4 (1.10-10.52**)*	**1.4 (0.44–4.17)**	3.4 (1.42–8.23)*
Unknown	5 (0.6)	3 (60.0)	2 (40.0)	0	0	0	2 (100.0)				
**Strain Family**											
Beijing	576 (66.7)	92 (16.0)	505 (84.0)	88 (18.2)	60 (12.4)	43 (8.9)	293 (60.5)	1.72 (1.15–2.56)*	4.5 (2.55–7.84)*	4.8 (2.49–9.27)*	15.8 (9.95–25.11)*
Non-Beijing	288 (33.3)	144 (50.0)	156 (50.0)	80 (55.6)	21 (14.6)	14 (9.7)	29 (20.1)	1	1	1	1

*P-value < 0.05

### Factors associated with clustering

**Fig. 1:**
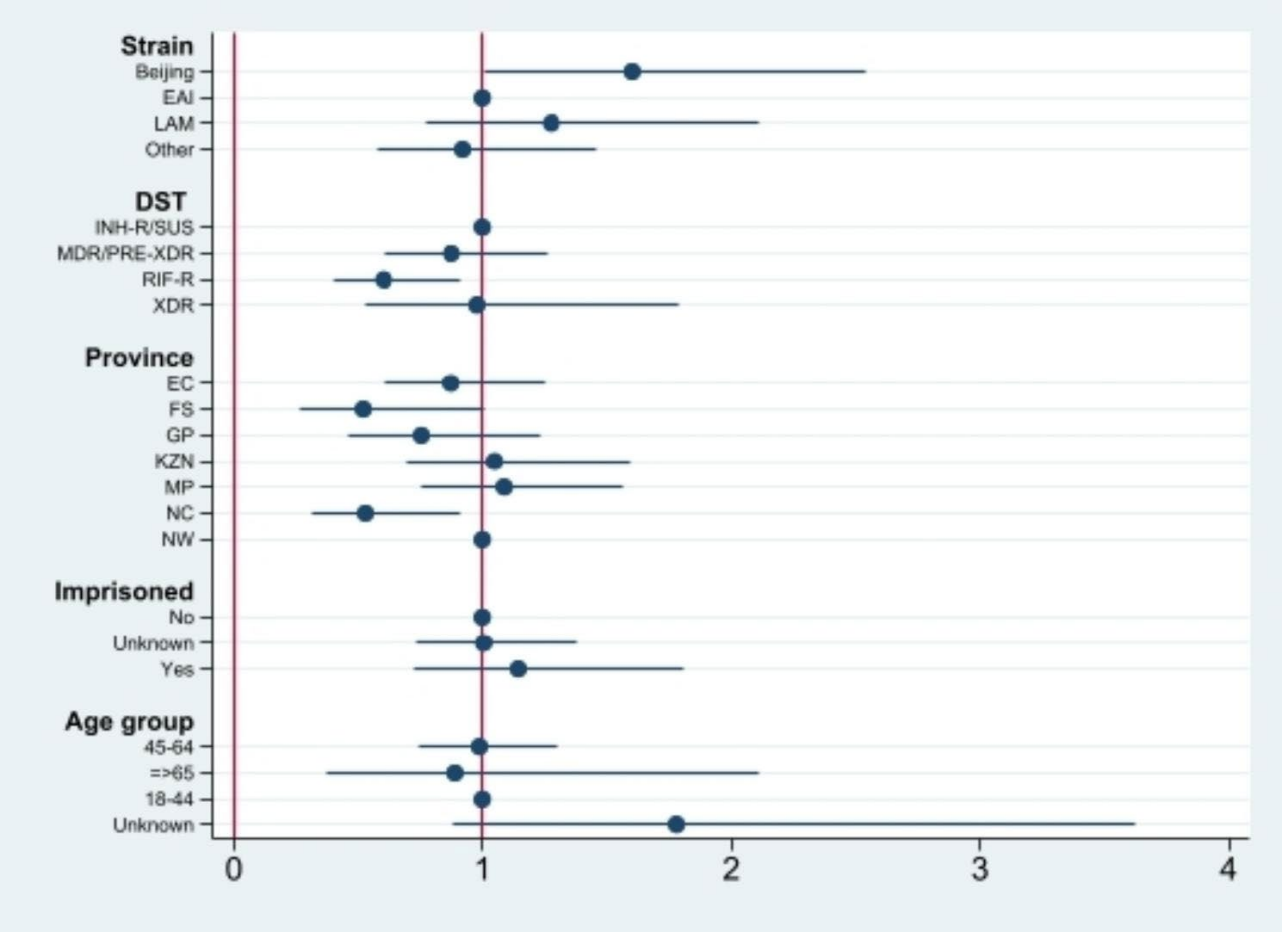
Coefficient plots of adjusted odds ratios with 95% confidence intervals from multinomial logistic regression analysis (Cluster = 2–5 cases)

**Fig. 2:**
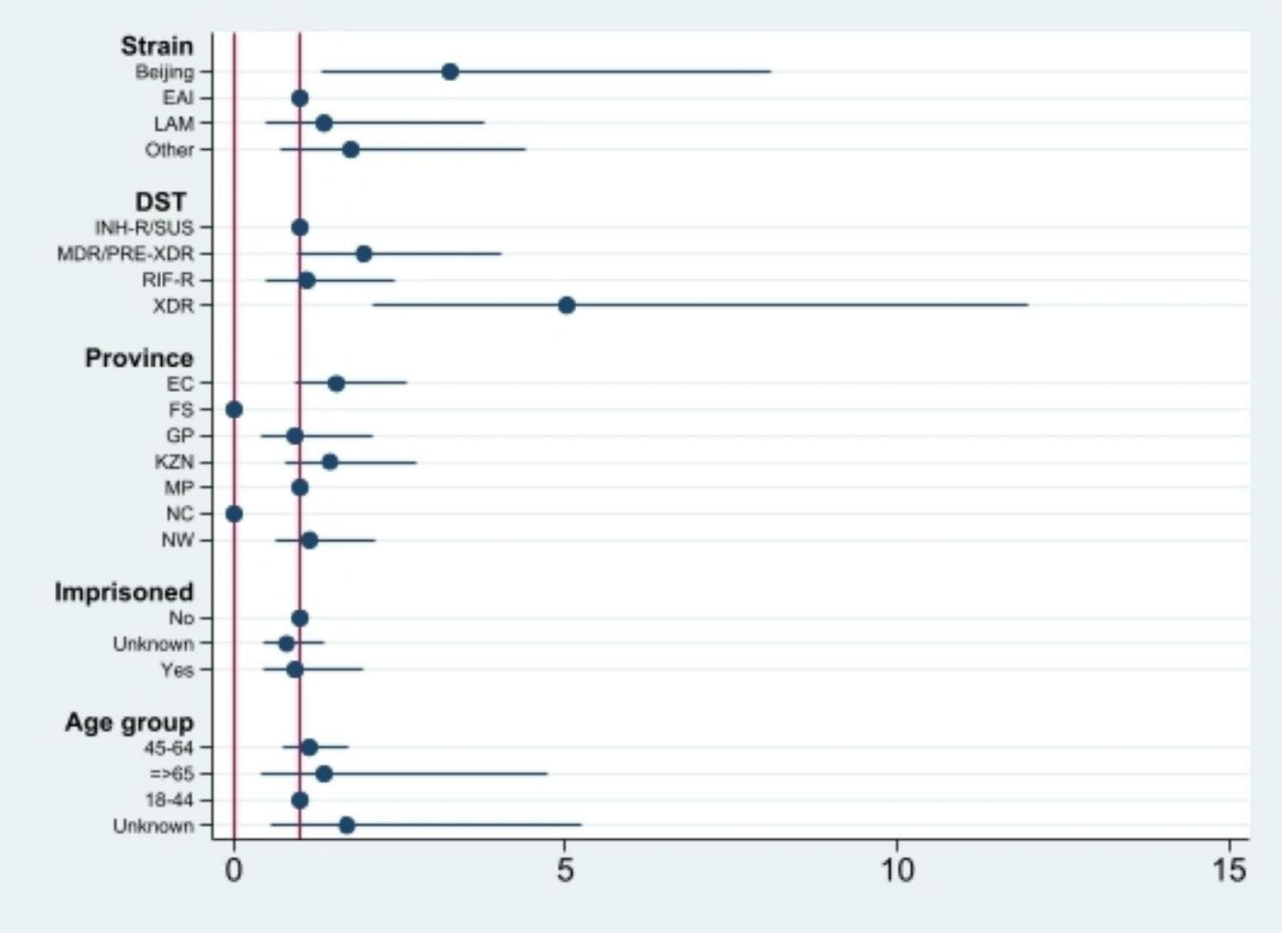
Coefficient plots of adjusted odds ratios with 95% confidence intervals from multinomial logistic regression analysis (Cluster = 6–10 cases)

**Fig. 3:**
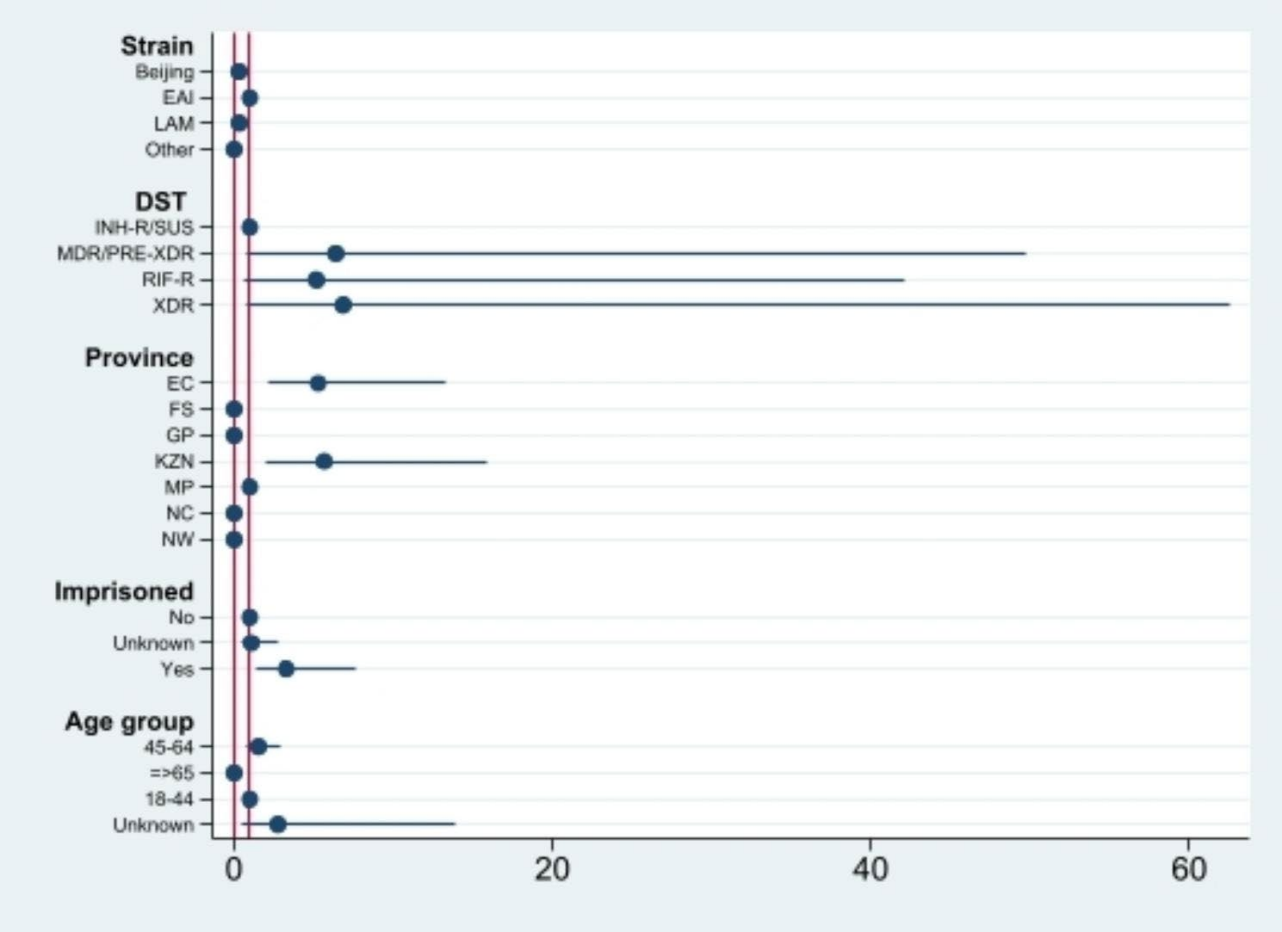
Coefficient plots of adjusted odds ratios with 95% confidence intervals from multinomial logistic regression analysis (Cluster = 11–25 cases)

**Fig. 4:**
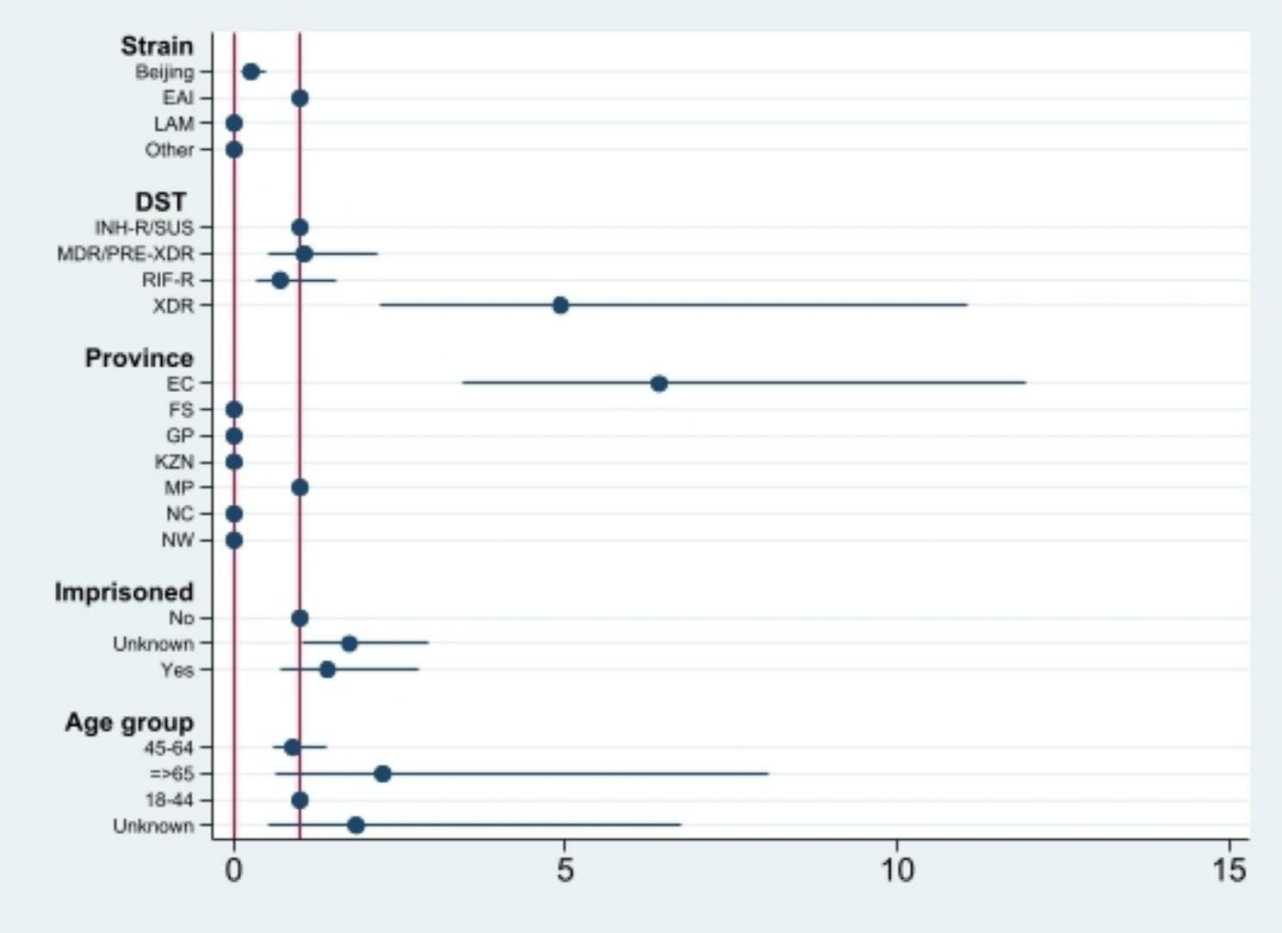
Coefficient plots of adjusted odds ratios with 95% confidence intervals from multinomial logistic regression analysis (Cluster = ≥ 26 cases)

Single-variable multinomial logistic regression analysis of cases from the seven surveillance sites are shown in Table 2; Figs. 1, 2, 3 and 4. Factors which were significantly associated after adjustment in the univariate analysis were included in the final multivariable model (Table 2). Patients in the 11–25 and with ≥ 26 isolates/cluster group were more likely to be infected by Beijing family (OR = 0.32, 95% CI 0.12–0.82 and OR = 0.23, 95% CI 0.12–0.46, respectively), having XDR-TB (OR = 5.08, 95% CI 2.26–11.40), living in EC (OR = 5.14, 95% CI 2.07–12.76 and OR = 6.53, 95% CI 3.46–12.35, respectively) or KZN (OR = 5.52, 95% CI 2.00-15.33) provinces, and having history of imprisonment (OR = 3.24, 95% CI 1.39–7.51). Individuals living in NC (OR = 0.51, 95% CI 0.29–0.87) or infected with RIF-R TB (OR = 0.60, 95% CI 0.40–0.91) were less likely to belong in a cluster > 5 isolates/cluster group (Table 2). However, being HIV positive, being previously treated, smear grading 2 + and 3+, having MDR-TB were associated with large or very large clusters only in the univariate analysis.

Multivariate analysis was not performed for WC as there was no risk-factors data (questioner data). In addition, some of the numbers in the available data for demographics and laboratory were small, therefor the confidence interval of the regression model was too wide making the analysis not meaningful. In the univariate analysis, having smear grading 2 + was significantly associated with cluster size n = 11–25. Patients infected with MDR and XDR TB are more likely to be in cluster ≥ 26 isolates/cluster group or cluster size n = 6–10. The odds for patients infected with Beijing was 15.8 times more likely to be in cluster ≥ 26, while only 1.72 times more likely to be in small cluster (2–5 cases) (Table 3).

## Discussion

The DR-TB epidemic has been attributed to several drivers, including environmental, social, and host-related risk-factors that promote transmission. In high-burden settings such as SA, considerable demographic and geographic heterogeneity in DR-TB transmission exists, implying that specific risk groups as well as high-burden areas might be prioritized for targeted intervention. Thus, in this study, we analyzed potential risk-factors for genotypic clustering in SA, during a five-year period, by comparing demographic, clinical and epidemiologic characteristics with cluster sizes. To our knowledge, this study is the largest that has been conducted in SA to assess risk-factors related to transmission.

The majority (81.6%) of the clusters identified in the study were small with few large and very large clusters identified mainly in districts from WC, EC and MP. Being part of a cluster suggests that *M. tuberculosis* was recently transmitted to the patient [1]. The size of clusters could depend on a number of factors related to the host, environment or differences in the strains themselves. In this study, specific cluster sizes were associated with either patient demographic, clinical, or epidemiological characteristics. Cases in either large or very large molecular clusters were more likely to have multiple risk-factors.

Variation in the distribution of clusters of DR-TB in different setting indicates different transmission dynamics. Living in Nelson Mandela Metro, EC was found to be a risk-factor in both univariate and multivariate analysis. EC province has the third highest number of people infected with DR-TB in SA [20]. The cases for the current study were from Port Elizabeth in the Nelson Mandela district, which is one of major city in the EC. TB is a major public health challenge in this district. One in 100 people is infected with TB and 90% of those diagnosed with TB are also co-infected with HIV and/or AIDS. The district have also a third highest rate of persons loss-to-follow on treatment [21]. Given these challenges the current TB control strategy need to implement rigorous TB and DR-TB surveillance systems for early case detection and treatment as well as improved transmission control measures.

In contrast, living in Mangauang, FS or Frances Baard, NC was associated with small cluster size in the univariate analysis, and the association remained significant for Frances Baard, NC in the multivariate analysis. Small cluster sizes may indicate small close contact transmission or reactivation of disease, emphasizing the importance of contact case investigations and infection control as the primary intervention in these areas.

In the univariate analysis, XDR-TB was associated with all cluster sizes (except small clusters). In the multivariate analysis, RR-TB was associated with small cluster sizes, while XDR-TB with medium and very large cluster sizes. DR-TB strains are more likely to be clustered than drug-sensitive cases due to the long treatment duration which might provide greater opportunity for transmission. Community-based active case-finding interventions is important, particularly in those settings where DR-TB cases are transmitted which may be attributed to community contacts. In addition, educating household and community members about DR-TB transmission, attitudes and prevention practices is needed.

In high-incidence setting, smear positivity is expected to be associated with clustering. Almost 90% of the TB transmission in the community is associated with sputum smear positive [22–24], as smear-positive patients often have more advanced disease and higher bacterial loads than those who are smear-negative. Also, higher smear grading could have a higher chance of transmitting disease and developing active TB among contacts than those with lower grading [23]. A meta-analysis study reported that compared to scanty, the sputum smear grading 2 + and 3 + were significant risk-factors in all the studies included [25]. In this study, smear grading 2 + and 3 + were associated with clustering only in univariate analysis. The lack of association between cluster size and smear grading might be due to the source cases being outside the sampled study population. The first cases for the large clusters are usually not identifiable. The majority of TB transmission in high-burden setting does not come from known contacts [26–28].

Being HIV positive was risk-factor for clustering (small and very large cluster size), but was not significant in the multivariate analysis. The role of HIV coinfection remains unclear, with some studies finding an increase in clustering of TB with HIV infection [12, 29] and others finding no association [30–33].

Workers of certain occupational sectors such as mining and healthcare sector are at particular risk for TB and transmission. In this study neither working in a mine or health sector were associated with clustering in the multivariate analysis. However, the majority (68%) of cases in this study were unemployed, which might limit the statistical power of this finding.

Large clusters of prevalent genotypes can become established in a certain area due to prolonged and uncontrolled transmission. The univariate analysis in this study showed infection with Beijing genotype are more likely to be medium cluster size (6–10 cases). The further multivariate analysis, however, showed association of Beijing genotypes with all cluster sizes. Beijing strains have wide spread distribution globally and are known to be associated with high clustering [34–37]. The majority of large or very large clusters in this study belonged to Beijing family, which suggests that these strains might have greater transmissibility [37].

Multivariate analysis was not performed for districts in WC, as there was no questionnaire data available. In the univariate analysis, smear positivity was a risk-factor for medium and large cluster sizes. The odds for patients infected with MDR was 7.7 times more likely to be in very large cluster (≥ 26 cases). The odds for Beijing genotype was 15.8 times more likely to be in cluster ≥ 26 as compared to only 1.49 times more likely to be in small cluster (2–5 cases). The Beijing genotype is endemic strain in WC. It was linked to an outbreak of MDR-TB at a school [38] and a subgroup of the Beijing family of strains (R220 genotype) were identified as commonly transmitted DR-TB strains in the province [39].

The study had a number of limitations. First, there is a selection bias in the study population because only culture positive samples in selected districts were included. Also, the study is a sentinel surveillance included only patients who accessed health care and consented to provide second sputum sample, thus patients who did not consent, undiagnosed and/or died in the community would not be included. As a result, our findings may not be generalizable to the entire SA population. Second, we were not able to obtain risk-factors data for all enrolled participants. Third, sample collection in the different provinces occurred during different time periods due to implementation considerations (approvals, logistics etc.), which could have impacted clustering analysis. Areas that had shorter sampling durations may have missed transmission events and underestimated clustering. Fourth, the clusters in study were not supported by contact investigations to confirm the linkages between clustered isolates using epidemiological data. Lastly, the possibility of overestimating clustering and recent TB transmission-rates is possible considering that the basis of the clustering analysis was done using traditional typing, whereas WGS could have offered a better resolution of strains and further discrimination between individuals in clusters. Despite these limitations, our study provides important information on risk-factors that might be contributing to the high DR-TB transmission in SA.

## Conclusion

Sociodemographic, clinical and bacterial risk-factors influenced rate of *M. tuberculosis* genotypic clustering. Hence, high-risk groups and hotspot areas for clustering in EC, WC, KZN and MP should be prioritized for targeted intervention to prevent ongoing DR-TB transmission.

## Data Availability

The datasets used and/or analysed during the current study are available from the corresponding author on reasonable request.

## List of abbreviations

DR-TB: Drug Resistant Tuberculosis
SA: South Africa
RR: Rifampicin-Resistant
MIRU-VNTR: mycobacterial interspersed repetitive-units-variable-number tandem repeats
LAM: Latin American and Mediterranean
EAI: East-African-Indian
H: Haarlem
*M. tuberculosis*: *Mycobacterium tuberculosis*
WGS: Whole Genome Sequencing
CTB: Center for TB
NICD: National Institute for Communicable Diseases
WC: Western Cape
EC: Eastern Cape
NW: North West
KZN: Kwa-Zulu Natal
GP: Gauteng
NC: Northern Cape
FS: Free State
MP: Mpumalanga
MDR: Multidrug Resistant
XDR: Extensively Drug Resistant
n: Number
HIV: Human Immunodeficiency Virus
INH: Isoniazid
RIF: Rifampicin
FLQ: Fluoroquinolone
AMK: Amikacin
KAN: Kanamycin
CAP: Capreomycin
DST: Drug susceptibility testing
IQR: interquartile range
